# Supplementary material for: Distinguishing the activity of adjacent somatosensory nuclei within the brainstem using 3T fMRI
Source: Imaging Neurosci (Camb). 2025 May 12;3:imag_a_00581. doi: 10.1162/imag_a_00581 (PMC12319763; doi:10.1162/imag_a_00581)
Supplement: Supplementary Material [file imag_a_00581-supp.pdf]

Supplementary Material

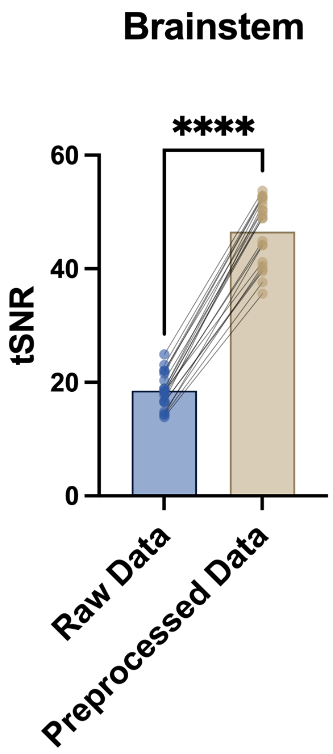

Supplementary Figure 1. Temporal signal-to-noise ratio (tSNR) before and after data preprocessing.
We found a significant increase in the temporal SNR in the whole brainstem mask following preprocessing (beige, mean = 46.53, s.e.m = 1.30) compared to the raw data (blue bar, mean = 18.50, s.e.m = 0.69); $t(19) = 33.56$, $p < .0001$, Cohen's $d = 7.51$. Statistical significance is denoted by: **** = $p < .0001$.

Post hoc sensitivity and generalisation analyses

*To determine whether our study had sufficient power to detect the observed effects in the brainstem, we conducted a post hoc sensitivity analysis using G*Power (Faul et al., 2007). For repeated measures ANOVA with a sample size of N = 20 and $\alpha = 0.05$, the sensitivity analysis indicated that we could detect an effect size of $\eta^2 = 0.08$ with 80% power. This effect size falls within the medium range based on conventional thresholds (Cohen, 1992). The effect sizes for the spinal trigeminal ($\eta^2 = 0.629$) and left cuneate ($\eta^2 = 0.562$) univariate ROI analyses were larger than this threshold, suggesting that the sample size was adequate to detect the observed effects (Table 1). However, we obtained a small effect size for the right cuneate univariate ROI analysis (W = 0.243), suggesting that a larger sample size may have benefited the comparison made for this ROI. Furthermore, the decoding of body-part selective activity has not been conducted in the brainstem before, which strongly limits our ability to calculate the sample size based on previous literature. We, therefore, explored the generalisation of our SVM decoder as a function of sample size.

Following the approach of Klement et al. (2008), we evaluated the cross-fold leave-one-subject-out error rate, training with randomised subject selections to mitigate subject selection bias. The sample size was systematically increased from 2 to 20 subjects in increments of 2, with each iteration repeated 100 times. Using a best-fit one-phase exponential model, we found that the error rate plateaued in the brainstem ROI at approximately N=10 subjects (plateau = 0.495 [95% CI: 0.488 to 0.503], R^2 = 0.641, τ = 1.321; Supplementary Figure 2). Additionally, we verified that the exclusion of left-handed participants did not alter the main results of the brainstem analyses (Left Cuneate ROI: $F(2,38) = 19.07$, $p < .0001$, $\eta^2 = 0.544$; Right Cuneate ROI: $X^2(2) = 6.71$, p = .035, W = 0.14; Brainstem Classification: mean accuracy = 50.5% (s.e.m = 3.31, p = < .001).

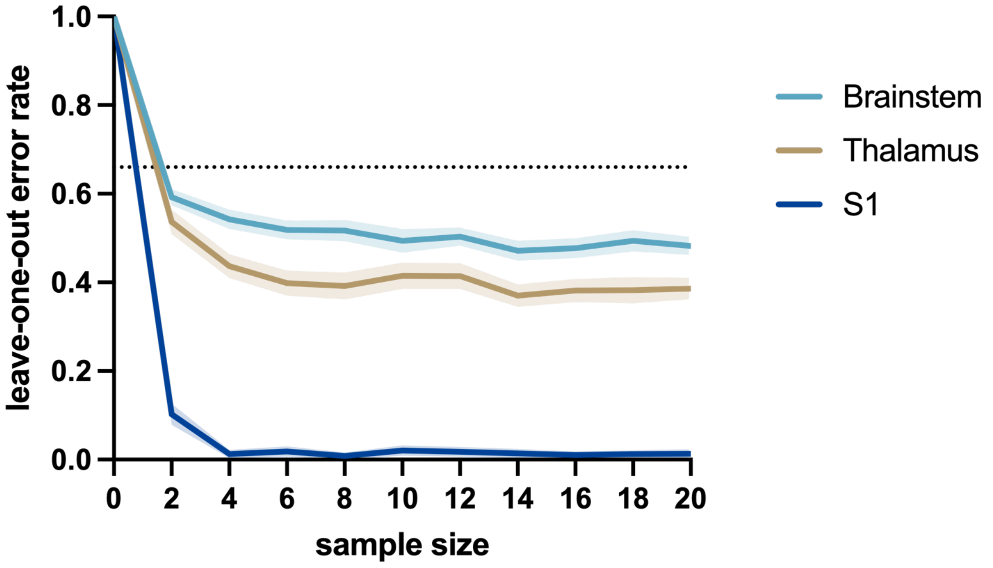

Supplementary Figure 2. Cross-fold leave-one-out error rate as a function of sample size and region of interest (ROI).
The solid lines represent the mean error rates across 100 random trials with the 95% confidence intervals shaded. Error rates plateaued for each ROI: brainstem ROI (light blue) at approximately N=10 subjects (plateau = 0.495 [95% CI: 0.488 to 0.503], R^2 = 0.641, τ = 1.321), thalamus ROI (beige) at N=6 (plateau = 0.392 [95% CI: 0.382 to 0.401], R^2 = 0.631, τ = 1.424), and S1 ROI (dark blue) at N=4 (plateau = 0.013 [95% CI: 0.010 to 0.017], R^2 = 0.959, τ = 0.826). The dashed line represents 1 – chance-level accuracy.

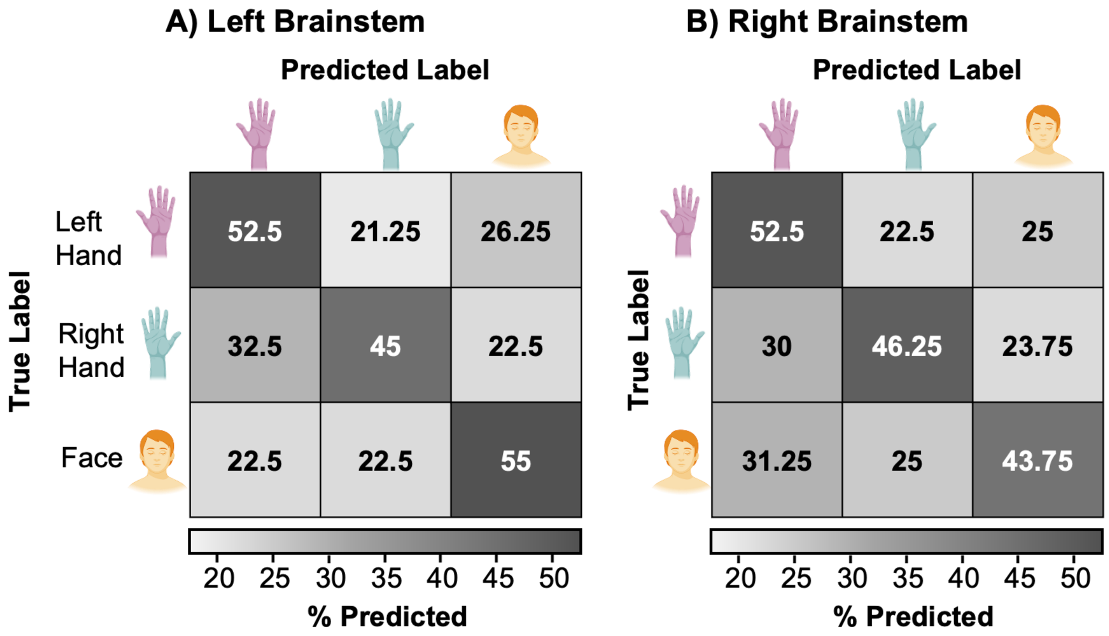

Supplementary Figure 3. Ipsilateral hand and face activation patterns can be distinguished within the brainstem using multivariate classification analyses.
We trained linear support vector machines to classify voxel-wise activity patterns across participants in anatomical masks of the left (A) and right brainstem (B). Each confusion matrix depicts the percentage of correct and incorrect classifications for the left-hand, right-hand, and face conditions (rows = true labels, columns = predicted labels). The mean multiclass classification accuracies for the left brainstem (50.8%, s.e.m. = 2.89) and the right brainstem (47.5% s.e.m. = 2.50) were both significantly above chance-level (33.3%; p = <.001).
